# In Vitro Anti-*Toxoplasma* Activity of Extracts Obtained from *Tabebuia rosea* and *Tabebuia chrysantha*: The Role of β-Amyrin

**DOI:** 10.3390/molecules29050920

**Published:** 2024-02-20

**Authors:** Maria Camila Cardona-Trujillo, Francisco Javier Jiménez-González, Luz Angela Veloza, Juan Carlos Sepúlveda-Arias

**Affiliations:** 1 Grupo Infección e Inmunidad, Facultad de Ciencias de la Salud, Universidad Tecnológica de Pereira, Pereira 660003, Colombia; camila.cardona@utp.edu.co; 2 Grupo Polifenoles, Facultad de Tecnologías, Escuela de Química, Universidad Tecnológica de Pereira, Pereira 660003, Colombia; jjimenez@utp.edu.co (F.J.J.-G.); lveloza@utp.edu.co (L.A.V.)

**Keywords:** anti-*Toxoplasma* activity, *Toxoplasma gondii*, Bignoniaceae, *Tabebuia chrysantha*, *Tabebuia rosea*, β-amyrin

## Abstract

Toxoplasmosis is a parasitic disease caused by the protozoan *Toxoplasma gondii* that is highly prevalent worldwide. Although the infection is asymptomatic in immunocompetent individuals, it severely affects immunocompromised individuals, causing conditions such as encephalitis, myocarditis, or pneumonitis. The limited therapeutic efficacy of drugs currently used to treat toxoplasmosis has prompted the search for new therapeutic alternatives. The aim of this study was to determine the anti-*Toxoplasma* activity of extracts obtained from two species of the genus *Tabebuia*. Twenty-six extracts, 12 obtained from *Tabebuia chrysantha* and 14 from *Tabebuia rosea*, were evaluated by a colorimetric technique using the RH strain of *T. gondii* that expresses β-galactosidase. Additionally, the activity of the promising extracts and their active compounds was evaluated by flow cytometry. β-amyrin was isolated from the chloroform extract obtained from the leaves of *T. rosea* and displayed important anti-*Toxoplasma* activity. The results show that natural products are an important source of new molecules with considerable biological and/or pharmacological activity.

## 1. Introduction

*Toxoplasma gondii* is an obligate intracellular parasite belonging to the phylum Apicomplexa, which causes the most common parasitic zoonosis worldwide [1,2]. It is known to be the causative agent of toxoplasmosis, a disease that affects approximately 1–3 billion people [3].

The sexual stages of *T. gondii* occur in members of the Felidae family, and for this reason, they are considered definitive hosts, while warm-blooded animals, including sheep, goats, rodents, pigs, and birds, are intermediate hosts [4]. Transmission to intermediate hosts, including humans, can result from the ingestion of tissue cysts present in raw or undercooked meat from infected animals, raw vegetables, and water contaminated with *T. gondii* oocysts. Moreover, vertical or transplacental transmission can occur from mother to fetus when tachyzoites cross the placenta during pregnancy and infect the fetus, causing congenital toxoplasmosis. The risk of vertical transmission increases with gestational age during pregnancy in mammals [5].

Parasitic infection causes various symptoms in birds, animals, marine mammals, and humans [6]. The clinical manifestations vary depending on the immune status of the host and the type of infecting strain. Generally, in immunocompetent individuals, there are no associated symptoms; however, in immunosuppressed individuals, several organs can be affected, promoting the development of encephalitis, myocarditis, or pneumonitis [2,7]. Similarly, the disease is considered serious in pregnant women because it can cause congenital toxoplasmosis, a neurological or ocular disease that can result in blindness [8].

Currently, the treatment regimens are based on the use of pyrimethamine, trimethoprim, and sulfadiazine. These drugs inhibit the enzymes dihydrofolate reductase (DHFR) and dihydropteroate synthetase (DHPS), which are involved in the synthesis of DNA in the parasite (folic acid pathway) [9]. Other medications, such as cotrimoxazole, have also been used, and steroids have been added to the treatment regimen for ocular toxoplasmosis [10,11]. However, therapeutic failures associated with current treatment regimens have been reported, which are due to individual factors such as intolerance and malabsorption, as well as the development of parasite resistance [12,13]. Due to therapeutic failures and the limited action of the available drugs, it is necessary to search for alternative sources for the development of new effective and safe drugs for the treatment of toxoplasmosis.

Natural products have played a very important role in developing therapeutic drugs due to the novel structural variety and large number of bioactive molecules they contain. Between 1981 and 2014, 221 chemical compounds were approved as anti-infective drugs worldwide, of which 148 came from natural products, representing approximately 67% of the total number of approved compounds [14]. The importance of natural products as a new source of molecules with anti-*Toxoplasma* activity has been reported [9].

*Tabebuia*, a large genus within the Bignoniaceae family, includes hundreds of trees found in the intertropical zone of America [15]. This genus has a wide phytochemical diversity, and between 1967 and 2018, more than 163 natural compounds were isolated, including iridoids, phenolic acids, phenylpropanoids, flavonoids, quinones, naphthoquinones, and triterpenes. Quinones and naphthoquinones are the most frequently isolated compounds and show various pharmacological properties, including cytotoxic, antioxidant, fungicidal, and analgesic properties. Therefore, *Tabebuia* species are a promising source of new compounds with great bioactive potential [16]. *Tabebuia rosea* has been well-studied and is frequently used to treat skin conditions. An iridoid isolated from the bark of *Tabebuia rosea* has been shown to have antimalarial properties [17,18]. Moreover, studies have shown that *Tabebuia chrysantha* has antitumor activity, which is attributable to its naphthoquinone and polyphenol components [19]. Similarly, various anti-infective activities of extracts obtained from plants of the genus *Tabebuia* have been evaluated, showing promising results [15]. Therefore, the aim of this study was to evaluate the in vitro anti-*Toxoplasma* activity of extracts obtained from the leaves and inner bark of *Tabebuia rosea* and *Tabebuia chrysantha* and the active component isolated from *T. rosea* (β-amyrin).

## 2. Results

### 2.1. Preliminary Phytochemical Analysis

The preliminary phytochemical analysis of the extracts prepared from the inner bark and leaves of *T. rosea* showed the presence of flavonoids, lignans, terpenes, aldehydes, ketones, and unsaturated fatty acids. Moreover, the preliminary phytochemical analysis of the extracts prepared from the inner bark and leaves of *T. chrysantha* showed the presence of lignans, coumarins, terpenes, sterols, iridoids, triterpenes, saponins, and unsaturated fatty acids in all of the extracts. The results are supplied as Supplementary Material (Tables S1–S4).

### 2.2. Effect of Extracts on Cell Viability

When the effect of different concentrations of the extracts on the viability of human foreskin fibroblasts (HFF-1) was evaluated (from 50 to 6.25 µg/mL), it was found that the chloroform extract obtained from the inner bark of *T. rosea* reduced the percentage of viability of HFF-1 cells (83.3%) at a concentration of 12.5 µg/mL; however, this difference was not statistically significant compared with untreated cells. At concentrations lower than 25 µg/mL, the ethyl acetate extracts obtained from the inner bark of *T. rosea* and the *n*-hexane, ethyl acetate, *n*-butanol, and water extracts prepared from the leaves of *T. rosea* did not affect cell viability (Table 1). Regarding the effect of the extracts obtained from *T. chrysantha*, it was found that the extracts in *n*-hexane, ethyl acetate, and *n*-butanol obtained from the inner bark, as well as the extracts in chloroform, ethyl acetate, and *n*-butanol obtained from the leaves, did not significantly affect cell viability (Table 2).

### 2.3. Screening of Extracts with Anti-Toxoplasma Activity

The inhibition of *T. gondii* proliferation induced by the extracts prepared from *T. rosea* and *T. chrysantha* was evaluated using the β-galactosidase assay. The extracts were evaluated at concentrations of 20, 10, 5, and 1 µg/mL. As a positive control, a mixture of pyrimethamine (0.3 µg/mL) and sulfadiazine (93.75 µM) was used. Of the 26 extracts evaluated, three showed an inhibitory effect on the proliferation of *T. gondii*. The chloroform extracts obtained from the leaves and inner bark of *T. rosea* and the chloroform extract obtained from the leaves of *T. chrysantha* significantly affected the proliferation of *T. gondii*, as shown in Table 3 and Table 4. The results indicate that the inhibitory effect of the extracts at concentrations of 10 and 20 μg/mL was similar to that of the sulfadiazine–pyrimethamine mixture (2.745 ± 0.29).

### 2.4. Determination of Anti-Toxoplasma Activity by Flow Cytometry

**Figure 1:**
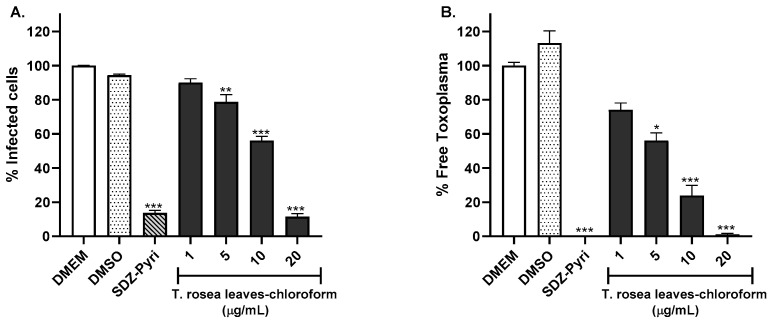
Evaluation of the anti-*Toxoplasma* activity of the chloroform extract prepared from *T. rosea* leaves by flow cytometry. The percentage of infected cells (**A**) and the percentage of extracellular or free parasites (**B**) were evaluated. DMEM and the sulfadiazine (93.75 µM)–pyrimethamine (0.3 µg/mL) mixture (SDZ-Pyri) were used as negative and positive controls, respectively. DMSO was also used as a vehicle control. The means were determined from four values obtained in three independent experiments (Kruskal–Wallis test), * *p* < 0.1, ** *p* < 0.01, *** *p* < 0.001 compared to the negative control.

The activity of the promising extracts was evaluated using the RH-GFP strain of *T. gondii*. The extracts were evaluated at concentrations of 1, 5, 10, and 20 µg/mL. Dulbecco’s Modified Eagle’s Medium (DMEM), dimethyl sulfoxide (DMSO), and a cocktail of pyrimethamine (0.3 µg/mL) and sulfadiazine (93.75 µM) were used as controls. Using the flow cytometry technique, it was possible to determine the percentage of infected cells and the percentage of parasites found at the extracellular level, as shown in Figure 1. The chloroform extract of *T. rosea* leaves at a concentration of 20 µg/mL considerably reduced the percentage of infected cells by up to 11.5% (Figure 1A, *p* < 0.001), achieving a greater effect than the antibiotic cocktail (13.6%). The results are related to the number of extracellular parasites, which significantly decreased with increasing extract concentration from 5 to 20 µg/mL (Figure 1B, *p* < 0.01 and *p* < 0.001, respectively).

**Figure 2:**
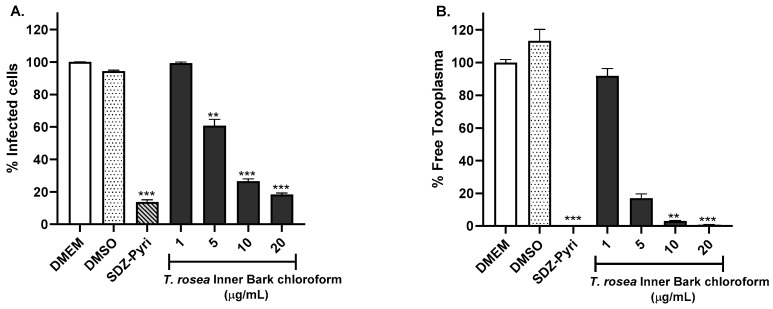
Evaluation of the anti-*Toxoplasma* activity of the chloroform extract prepared from *T. rosea* inner bark by flow cytometry. The percentage of infected cells (**A**) and the percentage of extracellular or free parasites (**B**) were evaluated. DMEM and sulfadiazine (93.75 µM)–pyrimethamine (0.3 µg/mL) mixture (SDZ-Pyri) were used as negative and positive controls, respectively. DMSO was also used as a vehicle control. The means were determined from four values obtained in three independent experiments (Kruskal–Wallis test), ** *p* < 0.01 and *** *p* < 0.001 compared to the negative control.

Figure 2 shows the results obtained from the evaluation of the chloroform extract prepared from *T. rosea* inner bark. The extract induced a greater inhibition of HFF-1 cell infection and *T. gondii* proliferation than that exhibited by the chloroform extract of *T. rosea* leaves. The concentration of 20 µg/mL notably reduced the percentage of infected cells to a value of 18.3% (Figure 2A), similar to the result obtained with the antibiotic cocktail used (13.6%). It was also observed that the extract mainly reduced free *Toxoplasma* since concentrations of 5, 10, and 20 µg/mL decreased the percentage of *Toxoplasma* by more than 80% (Figure 2B).

**Figure 3:**
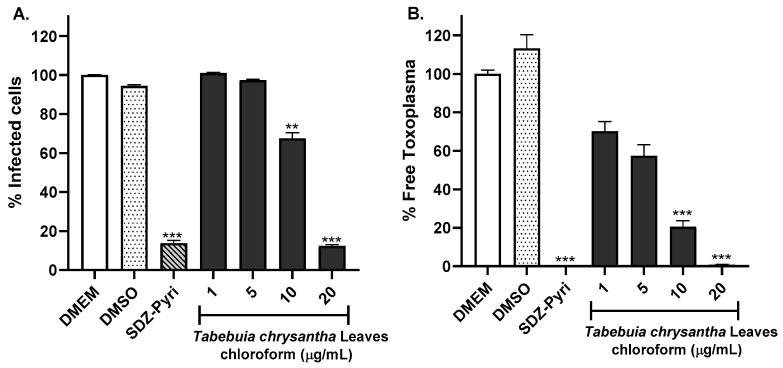
Evaluation of the anti-*Toxoplasma* activity of the chloroform extract prepared from the leaves of *T. chrysantha* by flow cytometry. The percentage of infected cells (**A**) and the percentage of extracellular or free parasites (**B**) were evaluated. DMEM and the sulfadiazine (93.75 µM)–pyrimethamine (0.3 µg/mL) mixture (SDZ-Pyri) were used as negative and positive controls, respectively. DMSO was also used as a vehicle control. The means were determined from four values obtained in three independent experiments (Kruskal–Wallis test), ** *p* < 0.01 and *** *p* < 0.001 compared to the negative control.

The chloroform leaf extract of *T. chrysantha* also inhibited the infection of HFF-1 cells (Figure 3). However, this effect was not as robust as the effects of the *T. rosea* extracts because the 5 µg/mL concentration of *T. chrysantha* leaf chloroform extract did not reduce the percentage of infected cells (Figure 3A). For this extract, the effect on the percentage of free *Toxoplasma* was greater than that on the percentage of infected cells (Figure 3B).

**Figure 4:**
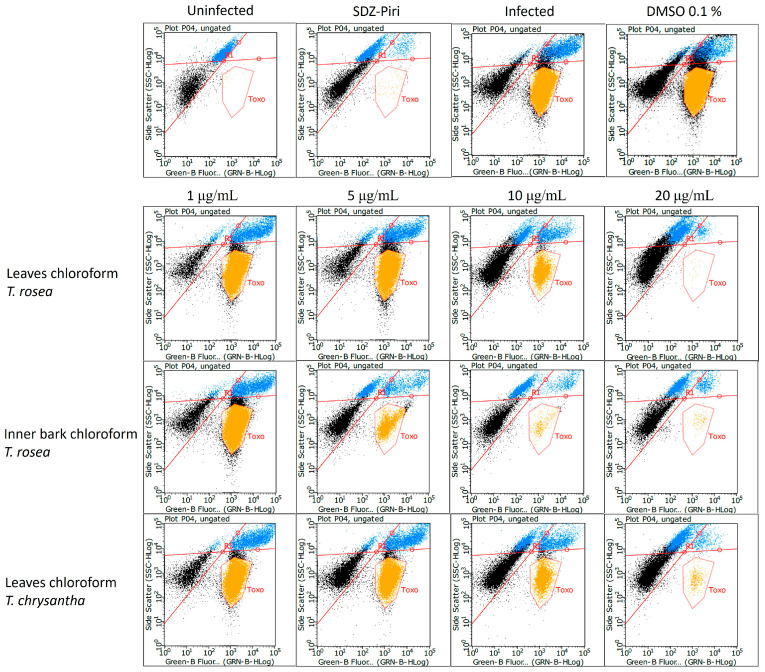
Evaluation of the anti-*Toxoplasma* activity of the chloroform extracts prepared from the leaves and inner bark of *T. rosea* and the chloroform extract prepared from the leaves of *T. chrysantha* by means of flow cytometry. The dot-plot result of a representative experiment is shown. Yellow color dots correspond to free (extracellular) *Toxoplasma*, and blue color dots represent *Toxoplasma* infected cells.

For the three promising extracts, a concentration-dependent effect was observed (Figure 4); the higher the concentration, the lower the percentage of infected cells and free *Toxoplasma*.

In Figure 4, the Y-axis is the size scale, and the fluorescence scale is on the X-axis. In the upper quadrant, the cells are shown, divided into two populations. The fluorescent cells on the right side are the cells infected with the GFP-RH strain of *T. gondii*. The cells on the left are those that the parasite did not infect. In the lower right are the extracellular tachyzoites, as they are smaller but fluorescent. The results indicate that as the extract concentration increases, the number of infected cells decreases, as well as extracellular tachyzoites, as previously indicated.

### 2.5. Determination of the Effect of a Fraction and Pure Compound on Cell Viability

**Figure 5:**
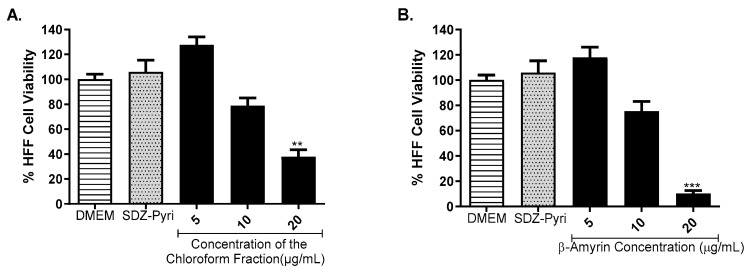
Effect of the chloroform fraction (**A**) and β-amyrin isolated from the chloroform fraction (**B**) from the chloroform extract of *T. rosea* on the HFF-1 cell viability method at concentrations of 20, 10, and 5 µg/mL. The MTT (3-[4,5-dimethylthiazol-2-yl]-2,5 diphenyl tetrazolium bromide) assay was used. Additionally, the effect of the sulfadiazine (SDZ, 93.75 µM) pyrimethamine (Pyri, 0.3 µg/mL) cocktail on the viability of HFF cells was evaluated. The means were determined from two values obtained in two independent experiments (Kruskal–Wallis Test), ** *p* < 0.01 and *** *p* < 0.001 compared to the negative control.

The cytotoxicity test was carried out for a fraction and a pure compound (β-amyrin) isolated from the chloroform extract prepared from *T. rosea* leaves, as it was one of those that presented the best anti-*Toxoplasma* activity, according to the screening carried out in the β-galactosidase assay. The concentrations of β-amyrin used were 20, 10, and 5 µg/mL. The results obtained are shown in Figure 5 and indicate that the concentration of the fraction and the compound, β-amyrin, that does not affect cell viability was 5 μg/mL. Additionally, it was observed that the sulfadiazine pyrimethamine mixture used as a positive control did not affect cell viability.

**Figure 6:**
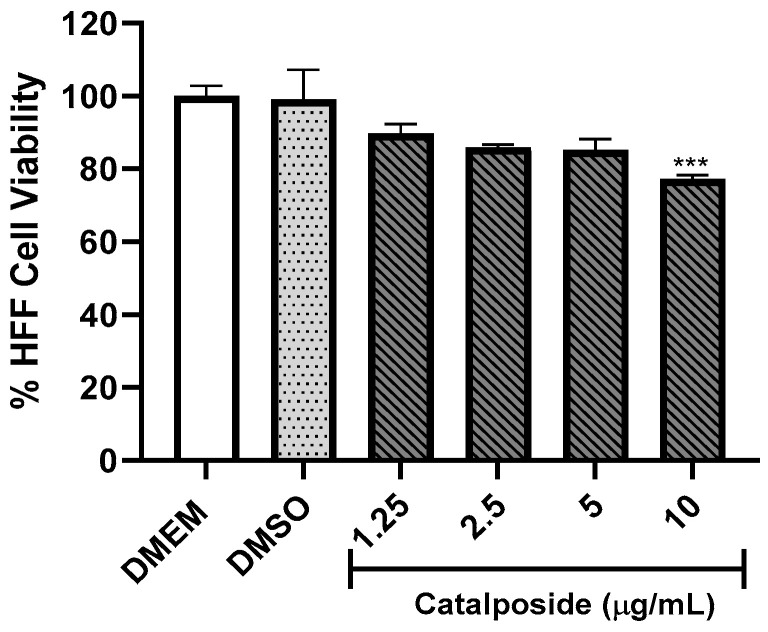
Effect of the catalposide isolated from the butanol extract of *T. rosea* inner bark on HFF-1 cell viability using the MTT assay. The concentrations of 10, 5, 2.5, and 1.25 µg/mL were used. The means were determined from two values obtained in two independent experiments (Kruskal–Wallis test), *** *p* < 0.001 compared to the negative control.

Another compound that was evaluated was catalposide, which was obtained from the butanol extract of the inner bark of *T. rosea* [20]. In this case, concentrations of 10, 5, 2.5, and 1.25 µg/mL were evaluated. The obtained results are shown in Figure 6 and indicate that the concentration of catalposide that does not affect cell viability was also 5 μg/mL.

### 2.6. Determination of the Anti-Toxoplasma Activity of the Fraction and Pure Compounds by Flow Cytometry

**Figure 7:**
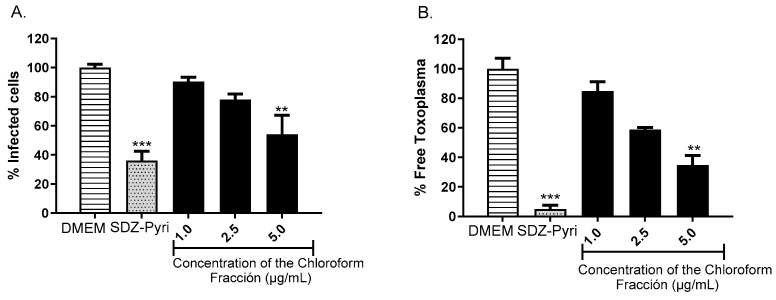
Evaluation of the anti-*Toxoplasma* activity of the fraction obtained from the chloroform extract of *T. rosea* leaves by flow cytometry. The percentage of infected cells (**A**) and the percentage of extracellular or free parasites (**B**) were evaluated. DMEM and the sulfadiazine (93.75 µM)–pyrimethamine (0.3 µg/mL) mixture (SDZ-Pyri) were used as negative and positive controls, respectively. The means were determined from three values obtained in three independent experiments (Kruskal–Wallis test), ** *p* < 0.01 and *** *p* < 0.001 compared to the negative control.

The activity of the fraction obtained from the chloroform extract of *T. rosea* leaves was evaluated using the RH strain of *T. gondii* that expresses the green fluorescent protein (GFP). The fraction was evaluated at concentrations of 1, 2.5, and 5 µg/mL, and DMEM and a cocktail of pyrimethamine (0.3 µg/mL) and sulfadiazine (93.75 µM) were used as controls. Using the flow cytometry technique, it was possible to determine the percentage of infected cells, as well as the percentage of parasites found at the extracellular level, as shown in Figure 7. The fraction evaluated at a concentration of 5 μg/mL significantly reduced the percentage of infected cells up to 54% (*p* < 0.01, Figure 7A); however, its effect was lower than that observed when using the SDZ-Pyri mixture (36%). Similar results were observed when evaluating the number of extracellular or free parasites after treatment (Figure 7B).

**Figure 8:**
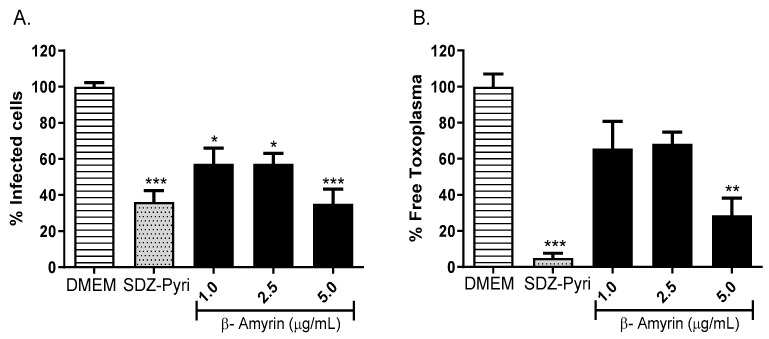
Evaluation of the anti-*Toxoplasma* activity of β-amyrin isolated from the chloroform extract of *T. rosea* leaves by flow cytometry. The percentage of infected cells (**A**) and the percentage of extracellular or free parasites (**B**) were evaluated. DMEM and the sulfadiazine (93.75 µM)–pyrimethamine (0.3 µg/mL) mixture (SDZ-Pyri) were used as negative and positive controls, respectively. The means were determined from three values obtained in three independent experiments (Kruskal–Wallis test), * *p* < 0.05, ** *p* < 0.01 and *** *p* < 0.001 compared to the negative control.

The activity of the β-amyrin isolated from the chloroform extract prepared from the leaves of *T rosea* was also evaluated using the RH-GFP strain of *T. gondii*. β-amyrin was tested at concentrations of 1, 2.5, and 5 µg/mL, and DMEM and a cocktail of pyrimethamine (0.3125 µg/mL) and sulfadiazine (93.75 µM) were used as controls. The percentage of infected cells was determined, as well as the percentage of parasites found at the extracellular level after treatment, as shown in Figure 8. The results indicate that β-amyrin significantly decreased the percentage of infected cells up to 35% at a dose of 5 μg/mL (*p* < 0.001, Figure 8A), an effect similar to that observed with the use of pyrimethamine–sulfadiazine (36%). This decrease in the percentage of infected cells was correlated with a significant decrease of 28% in the percentage of free or extracellular parasites (*p* < 0.01, Figure 8B).

**Figure 9:**
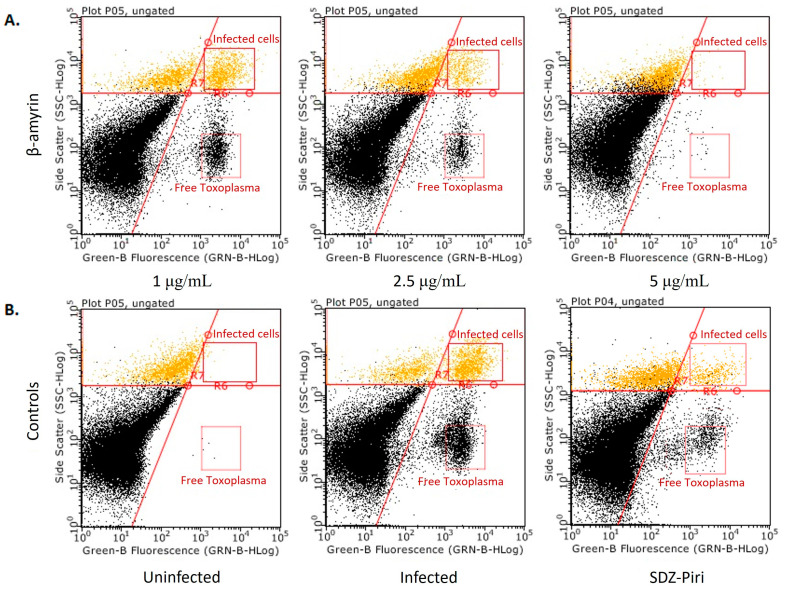
Evaluation of the anti-*Toxoplasma* activity of β-amyrin isolated from the chloroform extract of *T. rosea* leaves by flow cytometry. The dot plot from an independent test is shown. (**A**). Treatment with different β-amyrin concentrations ranging from 1 to 5 µg/mL. (**B**). Experimental controls (uninfected cells, infected cells, and treatment control). Yellow dots at the upper right side represent *Toxoplasma* infected cells.

In Figure 9A,B, the results obtained (dot plot) in a flow cytometry assay are shown. The results indicate that as the concentration of β-amyrin increases, the number of infected cells, as well as extracellular tachyzoites, decreases.

Finally, the activity of catalposide isolated from the *n*-butanol extract of *T. rosea* inner bark was also evaluated. However, this extract had no effect on the inhibition of infection and did not significantly reduce the percentage of free *Toxoplasma*.

Concerning the extracts and promising compounds, the therapeutic index was calculated based on the evaluated activity (Table 5). As expected, the chloroform extract obtained from the *T. rosea* inner bark showed the best therapeutic index.

## 3. Discussion

Natural products, especially the secondary metabolites produced by plants, are the source of most of the drugs used today. Natural products have been used as precursors for the development of new synthetic or semisynthetic drugs with antimicrobial activity, including antiprotozoal activity [15]. The use of natural products with anti-*Toxoplasma* activity has received increasing attention. In vitro models, and in some cases, in vivo models, have been used for the evaluation of plant extracts, showing promising results [21,22,23,24].

Plants of the genus *Tabebuia*, found mainly in the intertropical zone of the Americas, have been considered an important source of bioactive molecules such as naphthoquinones, quinones, phenols, and other molecules with anti-inflammatory, antioxidant, anti-proliferative, and antimicrobial activity [15,18,20,25,26,27]. Additionally, the results of ethnopharmacological studies highlight the use of plants from this genus for the treatment of various diseases [28]. Therefore, in the search for new sources of molecules with anti-*Toxoplasma* activity, the extracts obtained from the leaves and inner bark of *T. rosea* and *T. chrysantha* were evaluated in this study.

Preliminary phytochemical analysis showed the presence of coumarins in all extracts of the inner bark of *T. rosea*, as previously reported [25,26]. The presence of sesquiterpene lactones was not observed in any extract, which differs from the information reported by Jiménez-Gonzalez et al. [26]. Iridoids were found mainly in the methanol, hexane, chloroform, and ethyl acetate extracts, given the low polarity of these solvents. Terpenes were found in all of the extracts. It should be noted that the secondary metabolites that have been reported in the genus *Tabebuia* include flavonoids, iridoids, phenolic compounds, and naphthoquinones, compounds with high biological interest [28]. These compounds were present in the extracts evaluated in this study. Moreover, it is important to note that the metabolites found in a plant can change depending on conditions, such as the time of plant material collection, the type of soil in which the plant is cultivated, and the species [29]. Regarding the presence of antioxidant compounds, the compounds obtained in this study were in accordance with those that have been reported in the literature since the antioxidant activity of extracts obtained from *T. rosea* has been previously reported [26].

Cell viability tests showed that in general, the *n*-hexane, chloroform, ethyl acetate, *n*-butanol, and water extracts obtained from the leaves and inner bark of *T. rosea* and *T. chrysantha* did not affect the viability of HFF-1 cells when they were used at concentrations less than 25 μg/mL, so it was possible to evaluate the effect of these extracts on the proliferation of *T. gondii* using two different assays (colorimetric and flow cytometry). From the screening carried out using the RH strain of *T. gondii* that expresses the enzyme β-galactosidase, three promising extracts among the twenty-six evaluated were selected, including the chloroform extracts prepared from the leaves and inner bark of *T. rosea*, as well as the chloroform extract of *T. chrysantha* leaves. These compounds decreased the proliferation of *T. gondii* to numbers similar to those obtained with the pyrimethamine–sulfadiazine mixture, which was used as a positive control. It should be noted that the three extracts were obtained from the fractionation of the methanol extract with chloroform, so the nature of the molecules present in them is quite similar.

Similarly, the flow cytometry results showed that the extracts have a greater effect on the percentage of free *Toxoplasma* than on the percentage of infected cells. This finding suggested that the mechanism of action may be based on a direct cytotoxic effect on tachyzoites and not on the inhibition of cell infection.

The chloroform extract from the inner bark of *T. rosea* showed the greatest effect on the inhibition of infection and free *Toxoplasma* with an IC_50_ of 2.91 μg/mL; however, better results were obtained using the chloroform extract of *T. rosea* leaves and the RH-β1 strain. This is because, being transgenic strains, the virulence varies, and, therefore, the effect of the extract on the tachyzoites does as well. Additionally, the therapeutic indexes calculated from the flow cytometry results (Table 5) are the same and may even be higher than those of currently used drugs (pyrimethamine and sulfadiazine) since, in previous investigations, it was shown that sulfadiazine and pyrimethamine have lower TIs of ≤1 and ≤8, respectively [30,31].

Phytochemical studies of the extracts obtained from the leaves and inner bark of *T. rosea* show the presence of lignans, terpenes, aldehydes, and ketones; however, the chloroform extracts obtained from the leaves and bark of this species also showed the presence of iridoids, anthrones, quinones, and triterpenes, which is similar to what was previously found in the literature for extracts of leaves and inner bark of *T. rosea* [26]. Naphthoquinones, compounds identified in the vast majority of species from the genus *Tabebuia*, have been reported to inhibit the growth of *T. gondii* in vitro [32]. Although the mechanism of action of these compounds against *T. gondii* is unknown, it seems that a great variety of natural and synthetic naphthoquinones have antiprotozoal activity because they can generate reactive oxygen species (hydroxyl radical and superoxide anion), which can induce lipid peroxidation and inhibit the electron transport chain in the parasite [33,34,35]. Previous studies on *Tabebuia ochracea* ssp. *Neochrysanta* reported that the chloroform extract of this species has antimalarial activity in vitro against strains of *Plasmodium berghei*, a protozoan that causes malaria in rodents, which is associated with the furanonaphthoquinones present in the extract [36].

Various naphthoquinones isolated from species of the genus *Tabebuia*, such as *T. serratifolia*, *T. cassinoides*, and *T. ochracea*, have also been reported to have antileishmanial and trypanocidal activity in vitro [37,38], affecting, as previously mentioned, the electron transport chain in the parasite. It is important to note that lapachol [2-hydroxy-3- (3-methyl-2-butenyl)-1,4-naphthoquinone], abundant in the genus *Tabebuia* (*Handroanthus*) [39], has anti-*Trypanosome* [40], anti-*Leishmania* [41,42,43,44,45,46,47,48], and antimalarial activity [49].

This study and others have shown that the phytochemical profile of chloroform extracts obtained from the leaves and inner bark of *T. rosea* and *T. chrysantha* also include the presence of terpenoids, molecules that have been shown to have suitable antiprotozoal activity [50]. It is important to indicate that for the chloroform extracts obtained from the leaves and inner bark of *T. rosea*, an important antioxidant, anti-inflammatory, and antiproliferative activity has been reported in vitro [26].

Fractionation of the *T. rosea* chloroform extract was carried out, and the anti-*Toxoplasma* activity of this fraction was evaluated, as well as the β-amyrin isolated from the fraction TrH- CHF4- A [20,25], a pentacyclic triterpenoid. β-amyrin showed anti-*Toxoplasma* activity, significantly decreasing the intracellular proliferation of the parasite and, therefore, the number of infected cells, as well as the number of free tachyzoites in the culture medium, with an IC50 of 4.75 μg/mL and a TI of 15 (Table 5), a value much higher than that reported in the literature for antibiotics used as controls. To our knowledge, there are no reports in the literature regarding the anti-*Toxoplasma* activity of β-amyrin; however, there are studies that have reported the anti-*Toxoplasma* activity of pentacyclic triterpenoids such as ursolic acid and its derivatives [51,52]. It should be noted that terpenoids are considered promising compounds for the alternative treatment of various diseases of parasitic origin, among which are important drugs such as artemisinin [53,54]. This sesquiterpene lactone is known for its antimalarial activity; however, its mechanism of action is not clear, and it is believed that its activity is related to the production of reactive oxygen species [55]. Terpenoids, due to their great structural diversity, have various antiparasitic activities due to different mechanisms of action, including destabilization of the cell membrane, inhibition of essential enzymes in the parasite that trigger structural changes, arrest of the cell cycle, and finally, cell death [50].

A systematic review of the recent literature shows the importance of pentacyclic triterpenes and their derivatives in the search for new molecules with antiparasitic activity, which include α-amyrin and β-amyrin, among others [54]. Additionally, recent reports indicate that terpenoids have toxoplasmicidal activity, and their effect is focused on the reduction of extracellular tachyzoites since these types of compounds induce the apoptosis of tachyzoites by generating an imbalance in calcium levels and altering the mitochondrial membrane potential [56]. The characterization of phytochemical compounds within the extracts showed that the chloroform extracts were rich in terpenes, supporting previous reports. Moreover, the flow cytometry results indicated a direct cytotoxic activity on *T. gondii* tachyzoites.

The findings of this study show the potential of natural products in the search for new molecules with anti-*Toxoplasma* activity. Among these molecules, β-amyrin exerted a promising effect, although its in vitro activity was lower than that of the sulfadiazine–pyrimethamine combination. This pentacyclic triterpenoid or its derivatives could become new candidates for the treatment of toxoplasmosis; however, more studies would be needed to confirm its efficacy and to determine its mechanism of action. Other extracts with promising activity were identified in this study and should be further evaluated to determine the presence of new compounds with therapeutic potential against *T. gondii*.

## 4. Materials and Methods

### 4.1. Reagents

Analytical grade organic solvents were purchased from JT Baker (Phillipsburg, NJ, USA) and Mallinckrodt Baker (San Diego, CA, USA). Molecular-grade dimethylsulfoxide (DMSO 99.9%) and MTT (3-(4,5-dimethylthiazol-2-yl)-2,5-diphenyltetrazole bromide) were obtained from Sigma–Aldrich (Deutschland, Germany) and X-Gal (5-bromo-4-chloro-3-indolyl-β-D-galactopyranoside) was obtained from Sigma–Aldrich. Dulbecco’s Modified Eagle’s Medium (DMEM), Glutamax supplement, fetal bovine serum (FBS), trypsin, sodium pyruvate, and phosphate-buffered saline (PBS) were obtained from Gibco (Gaithersburg, MD, USA).

### 4.2. Plant Material and Preparation of Extracts

The leaves and stems of *Tabebuia rosea* (Bertol.) DC and *Tabebuia chrysantha* (JACQ) G. Nicholson were collected on the Campus of the Universidad Tecnológica de Pereira in May 2021. The collection and processing of the material was supported by the collection permission number 1133/2014, issued by the National Environmental Licensing Authority (Autoridad Nacional de Licencias Ambientales—ANLA) of Colombia.

The plant material was dried at room temperature and subsequently heated in an oven at 40 °C. Once dry, it was crushed in a grinder. After grinding up the leaves and the inner bark of the species of interest, approximately 800 g of crushed plant material was added to methanol at a 1:3 (*w*:*v*) ratio for extraction. The extraction process was carried out using an ultrasonic homogenizer for 2 h, followed by passive extraction. For the leaves of *T. chrysantha*, three extractions with dichloromethane were carried out, and the mixture was ultrasonicated for one hour, followed by passive extraction. Finally, repeated extractions were performed with methanol until exhaustion.

Once the extractions were complete, the extracts were dried by rotary evaporation in a water bath at 50 °C. For fractionation, approximately 50 g of the extracts of the inner bark and leaves of *T. rosea* and 25 g of the extracts of the inner bark and leaves of *T. chrysantha* were used. Once weighed, these were dissolved in 400 mL of distilled water, and a liquid–liquid extraction was carried out with solvents of increasing polarity: *n*-hexane, chloroform, ethyl acetate, and *n*-butanol. Subsequently, the extracts were concentrated in a rotary evaporator at reduced pressure and dried in an oven at 50 °C for subsequent analysis, as reported previously [26].

### 4.3. Preliminary Phytochemical Analysis

Preliminary phytochemical analysis was performed by selective derivatization reactions to characterize the secondary metabolites present in the methanol, *n*-hexane, chloroform, ethyl acetate (soluble and insoluble fractions), *n*-butanol, and water extracts. The characterization was carried out using thin-layer chromatography (TLC) in normal phase (silica) and reversed-phase (RP-18) with hexane–ethyl acetate (7:3) and water–isopropanol (7:3) elution systems, respectively. The chromatographic plates were developed with aluminum chloride (AlCl_3_, Sigma Chemical Co., Saint Louis, MO, USA), ferric chloride (FeCl_3_, Sigma Chemical Co., Saint Louis, MO, USA), and a mixture of vanillin–phosphoric acid (H_3_PO_4_) for the detection of flavonoids, phenols, and lignans. Potassium hydroxide (KOH, Merck, Darmstadt, Germany) in analytical grade ethanol was used for the detection of anthrones, quinones, and coumarins; a mixture of anisaldehyde–acetic acid–sulfuric acid (H_2_SO_4_) with vanillin–phosphoric acid (H_3_PO_4_) was used for the detection of iridoids; a mixture of anisaldehyde–acetic acid–sulfuric acid (H_2_SO_4_) with antimony chloride (SbCl_3_) was used for the detection of saponins and triterpenes; oleum (Sigma Chemical Co., Saint Louis, MO, USA) was used for the detection of sesquiterpene lactones; 2,4-dinitrophenylhydrazine was used for the determination of aldehydes and ketones; and the Liebermann–Burchard reagent was used for the detection of terpenes and steroids. The extracts and fractions showed a specific color when they reacted with the developing reagents for each test. The absence or presence of this color was taken as a negative (−) or positive (+) result for the presence of these phytochemical components.

### 4.4. Fractionation and Isolation of β-Amyrin

From the chloroform extract obtained from the leaves of *T. rosea*, chromatographic separation was carried out on a column packed with silica gel 60 using a step-by-step elution system until the fractions CHF1 (2 mg), CHF2 (13 mg), CHF3 (7 mg), CHF4 (2281 mg), CHF5 (131 mg), and CHF6 (962 mg) were obtained; each of the collected fractions was monitored by CCD [Hex/(CH_3_)_2_CO (7:3)] in the normal phase using Oleum as a developer. In this case, the CHF4 fraction showed a single spot at Rf = 0.47, which was intensely red with Oleum reagent, indicating the presence of sesquiterpene lactones and/or triterpenes.

Subsequently, the CHF4 fraction was separated by CC packed with silica gel in a reversed phase until the CHF4-A (471 mg), CHF4-B (50 mg), CHF4-C (31 mg), CHF4-D (8 mg), and CHF4-E (6 mg) fractions were obtained. The fractions were analyzed by TLC and developed with Oleum reagent, identifying the fractions CHF4-A and CHF4-C as promising fractions, which showed Rf stains of 0.5 and 0.32, respectively. The β-amyrin was isolated from the CHF4-A fraction using a semipreparative HPLC-DAD system (Hitachi-Merck) in reversed-phase (LiChrocart 250-10, LiChrospher 100; 10 µm, Merck, Darmstadt, Germany) by isocratic elution with H_2_O-ACN (70:30% *v*/*v*) containing 1% *v*/*v* CH_3_COOH.

**Figure 10:**
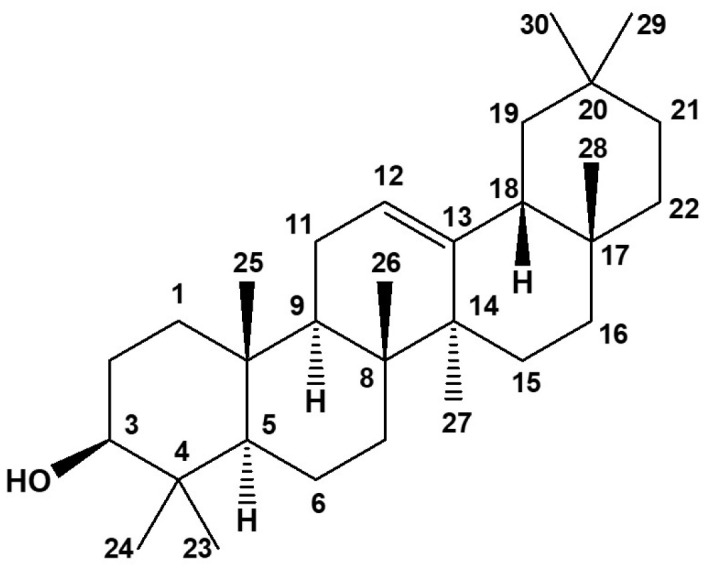
β-amyrin isolated from the chloroform extract obtained from the leaves of *T. rosea*.

Full assignments from the ^1^H and ^13^C NMR spectra were made through the use of ^1^H-^1^H COSY, HSQC, and HMBC experiments. All the experiments were performed on a 400 MHz Agilent spectrometer (125.6 MHz for ^13^C) using deuterated chloroform as solvent. β-amyrin showed an ^1^H NMR (CDCl_3_, 400 MHz) spectrum with characteristic signals at δ 5.25 (t, *J* = 3.6 Hz, 1H) and 3.21 (dd, *J* = 11.1, 4.9 Hz, 1H) for protons at H-12 (C-12 at 125.84 ppm) and H-3 (C-3 at 79.02 ppm), respectively, which were observed in the HSQC spectra. Additionally, signals characteristic of the presence of hydrogens at position H-5 at 0.72 ppm (dd, *J* = 11.6, 1.3 Hz, 1H) with C-5 at 55.19 ppm; H-9 at 1.49 ppm (d, *J* = 8.9 Hz, 1H) with C-9 at 47.88 ppm; and finally, the proton at H-11 at 1.91 ppm (dd, *J* = 8.9, 3.6, 6.3 Hz, 2H) with C-11 at 23.27 ppm. The ^13^C NMR (126 MHz, CDCl3) spectrum, together with the COSY, HSQC, and HMBC spectra, showed the presence of 29 signals corresponding to carbon atoms at δ 137.90 (C-13), 47.51 (C-18), 41.98 (C-14), 39.46 (C-8), 38.83 (C-4), 38.58 (C-1), 36.98 (C-22), 36.68 (C-10), 32.94 (C-7), 28.11 (C-23), 27.20 (C-15), 23.55 (C-30), 18.27 (C-6), 16.96 (C-26), 15.57 (C-25), and 15.45 (C-24). These results are similar to those previously reported in the literature [57]. Characteristic ^1^H NMR, ^13^C NMR, COSY, HSQC, and HMBC spectra are supplied as Supplementary Material. The structure of the β-amyrin isolated is shown in Figure 10.

### 4.5. Cell Culture

The human foreskin fibroblast HFF-1 cell line (ATCC) was used. The cells were cultured in DMEM supplemented with 10% FBS, 1% penicillin, streptomycin, neomycin (PSN), amphotericin B (2.5 µg/mL), and 1% sodium pyruvate. Trypsin was used to detach the cells from the culture flask. The cells were collected and resuspended in a supplemented culture medium for passage. The cells were cultured at a density of 1 × 10^4^ cells per well at 37 °C and 5% CO_2_ until reaching 90% confluence. The extracts were resuspended in DMSO and further diluted in the culture medium. Final concentrations lower than 0.1% were used to avoid affecting the viability of HFF-1 cells.

### 4.6. Parasites

The tachyzoites of the *Toxoplasma* strains RHβ1 and RH-GFP (kindly provided by Dr. Jorge Enrique Gómez-Marin, Universidad del Quindío) were propagated by infection of HFF-1 cells. The infected cells were cultured in DMEM supplemented with 2% FBS, 1% PSN, amphotericin B (2.5 µg/mL), and 1% sodium pyruvate. Once infected, the cells were incubated at 37 °C and 5% CO_2_. After cell lysis, the supernatant was centrifuged, and the tachyzoites were filtered through a 3 µm membrane to remove cells and cellular debris. The viability of the parasites was determined by the trypan blue method. The isolated parasites were resuspended in the corresponding medium for the inoculation of HFF-1 cells in the subsequent experiments.

### 4.7. Cell Viability Assay

The viability of HFF-1 cells was determined using the MTT method [58]. The cells were cultured at a density of 1 × 10^4^ cells per well in 96-well plates and incubated at 37 °C and 5% CO_2_. The extracts were evaluated at concentrations of 50, 25, 12.5, and 6.25 μg/mL. The fractions were used at concentrations of 20, 10, and 5 μg/mL, and the pure compounds were used at 20, 10, 5, 2.5, and 1.25 μg/mL. A solution of 0.3 µg/mL pyrimethamine and 93.75 µM sulfadiazine was used as a positive control. DMSO was used as the solvent control. The viability of cells treated with pyrimethamine–sulfadiazine was also evaluated.

### 4.8. Screening of Extracts with Potential Anti-Toxoplasma Activity

The anti-*Toxoplasma* activity of the 26 extracts was evaluated using the β-galactosidase colorimetric assay. HFF-1 cells were cultured in a 96-well plate at a density of 1 × 10^4^ cells per well and were allowed to grow to 90% confluence at 37 °C and 5% CO_2_. The medium was discarded, and each well was infected with 2 × 10^4^ tachyzoites of the RHβ1 strain and incubated for 6 h. After 6 h, the culture medium was removed, the cells were washed with PBS, and the extracts were added at concentrations of 20, 10, 5, and 1 µg/mL. The plates were incubated again for 48 h. As time passed, the medium was removed, and 25 µL of lysis solution (100 mM HEPES, 1 mM MgSO_4_, 0.1% Triton X-100, 5 mM dithiothreitol) was added for 15 min. Then, 25 µL of buffer (100 mM PBS pH 7.3, 102 mM β-mercaptoethanol, 9 mM MgCl_2_) was added, followed by 100 µL of X-Gal (5-bromo-4-chloro-3-indolyl-β-D-galactopyranoside), which was diluted to a concentration of 0.5 mg/mL in PBS. The plates were incubated for 15 h, after which the absorbance was measured at 630 nm in a Multiskan GO microplate spectrophotometer (Thermo Scientific, Waltham, MA, USA). The antibiotics pyrimethamine and sulfadiazine were added to infected cells as a positive control. Culture medium served as the negative control, and 0.1% DMSO served as the solvent control [59,60]. The concentrations of pyrimethamine and sulfadiazine were determined experimentally by means of three-dimensional curves, where it was found that at concentrations of 0.3 µg/mL pyrimethamine and 93.75 µM sulfadiazine, parasite viability was decreased to 43% without affecting the viability of HFF-1 cells.

### 4.9. Determination of Anti-Toxoplasma Activity by Flow Cytometry

The anti-*Toxoplasma* activity of the promising extracts, fractions, and pure compounds was evaluated using a *Toxoplasma gondii* strain that expresses green fluorescent protein (GFP). HFF-1 cells were cultured in a 24-well plate at a density of 6 × 10^4^ cells per well and allowed to grow to 90% confluence at 37 °C and 5% CO_2_. Each well was infected with 12 × 10^4^ tachyzoites of the RH-GFP strain and incubated for 4 h. After 4 h, the medium was removed, the cells were washed with PBS, and the extracts were added at concentrations of 20, 10, 5, and 1 μg/mL. The fractions and pure compounds were used at concentrations of 5, 2.5, and 1 μg/mL. After incubation for 48 h, the culture medium was removed, 200 μL of TrypLE™ Express was added and incubated for 3 min at 37 °C, then 600 μL of culture medium was used to perform well washes. The tubes were centrifuged for 5 min at 3500 rpm, the supernatant was removed, and 100 μL of 4% paraformaldehyde (PFA) was added for 10 min for fixation. Subsequently, 300 μL of PBS was added, and the samples were centrifuged again. Finally, the supernatant was removed, and the pellet was resuspended in 300 μL of flow cytometry buffer (2% FBS in PBS). The analysis was performed on a Guava EasyCyte (Merck) flow cytometer using InCyte software (version 3.1).

### 4.10. Statistical Analysis

The data obtained are the average of three independent tests in which each concentration of each extract was tested in triplicate, both for the cytotoxicity test and for anti-*Toxoplasma* activity. The data were analyzed and plotted with GraphPad Prism version 8.0.1 (GraphPad software, Boston, MA, USA). For the anti-*Toxoplasma* activity tests, a Kruskal–Wallis test was performed, with Dunn’s test as a post hoc test, to analyze the differences between the negative control and the activity of the extracts at different concentrations. Differences were considered significant when *p* < 0.05.

## 5. Conclusions

The results of this study indicate that the chloroform extracts obtained from the leaves and inner bark of *T. rosea*, the chloroform extract from the leaves of *T. chrysantha*, and β-amyrin isolated from the chloroform extract of *T. rosea* leaves have anti-*Toxoplasma* activity in vitro, reinforcing the importance of the study of plants in the genus *Tabebuia* to search for new molecules with promising biological activity.

## Figures and Tables

**Table 1:** Effect of *T. rosea* extracts on the viability of HFF-1 cells.

Part of the Plant	Extract	Percentage of Viable Cells (Mean ± SEM)			
		6.25 µg/mL	12.5 µg/mL	25 µg/mL	50 µg/mL
**Inner bark**	Methanol	101.1 ± 3.2	98.8 ± 4.6	96.9 ± 3.0	92.5 ± 2.8
	*n*-hexane	99.2 ± 2.7	95.6 ± 1.9	89.6 ± 2.4	84.2 ± 2.7
	Chloroform	93.1 ± 2.5	83.3 ± 2.3	68.1 ± 4.3	31.7 ± 9.5
	Insoluble ethyl acetate	104.0 ± 2.6	100.5 ± 2.2	102.0 ± 1.6	99.3 ± 2.1
	Soluble ethyl acetate	99.3 ± 2.7	101.8 ± 2.9	101.9 ± 3.3	102.5 ± 3.2
	*n*-butanol	90.7 ± 1.9	90.4 ± 2.5	92.1 ± 2.2	97.0 ± 3.0
	Aqueous	88.4 ± 1.5	89.9 ± 1.7	86.3 ± 5.2	94.4 ± 3.2
**Leaves**	Methanol	106.7 ± 1.3	102.8 ± 1.8	105.3 ± 1.9	107.9 ± 1.3
	*n*-hexane	106.9 ± 3.4	98.5 ± 2.2	95.7 ± 2.5	99.3 ± 4.1
	Chloroform	113.1 ± 2.0	109.5 ± 1.8	130.0 ± 3.1	58.8 ± 8.3
	Ethyl acetate 1	105.2 ± 2.8	105.2 ± 2.0	111.1 ± 1.9	113.5 ± 1.9
	Ethyl acetate 2	98.3 ± 2.9	98.6 ± 2.4	102.0 ± 2.1	107.2 ± 1.0
	*n*-butanol	95.5 ± 1.5	96.9 ± 1.0	103.6 ± 1.7	104.7 ± 2.8
	Aqueous	93.2 ± 1.4	95.5 ± 1.4	96.6 ± 2.1	100.2 ± 1.4

**Table 2:** Effect of *T. chrysantha* extracts on the viability of HFF-1 cells.

Part of the Plant	Extract	Percentage of Viable Cells (Mean ± SEM)			
		6.25 µg/mL	12.5 µg/mL	25 µg/mL	50 µg/mL
**Inner bark**	Methanol	98.3 ± 2.2	102.3 ± 3.1	99.1 ± 1.1	103.3 ± 2.0
	*n*-hexane	105.4 ± 2.2	109.3 ± 2.3	108.4 ± 2.1	106.1 ± 4.1
	Chloroform	102.7 ± 2.4	96.8 ± 1.7	92.0 ± 1.1	66.3 ± 2.8
	Soluble ethyl acetate	96.4 ± 2.0	99.1 ± 1.7	100.0 ± 3.7	102.5 ± 3.1
	Insoluble ethyl acetate	95.4 ± 1.8	94.6 ± 1.6	101.3 ± 1.5	107.9 ± 1.5
	*n*-butanol	100.3 ± 4.5	95.6 ± 1.2	94.0 ± 2.4	100.5 ± 1.9
	Aqueous	97.1 ± 2.5	98.0 ± 1.5	96.6 ± 1.5	96.8 ± 1.2
**Leaves**	Methanol	105.3 ± 3.2	98.8 ± 3.5	100.6 ± 2.9	129.1 ± 4.3
	Chloroform	98.2 ± 3.2	98.5 ± 1.9	112.6 ± 4.5	123.0 ± 2.9
	Ethyl acetate	105.9 ± 5.4	99.3 ± 2.7	100.8 ± 4.0	100.0 ± 3.4
	*n*-butanol	97.4 ± 4.1	91.6 ± 2.7	92.2 ± 2.6	101.5 ± 1.9
	Aqueous	96.4 ± 3.3	89.7 ± 3.1	93.1 ± 4.8	88.8 ± 4.7

**Table 3:** Effect of extracts prepared from *T. rosea* on the proliferation of *T. gondii* in HFF-1 cells.

Part of the Plant	Extract	Percentage of Proliferation (Mean ± SEM)			
		1 µg/mL	5 µg/mL	10 µg/mL	20 µg/mL
**Inner bark**	Methanol	85.4 ± 8.8	98.2 ± 5.7	104.5 ± 8.9	106.4 ± 9.8
	*n*-hexane	103.0 ± 11.1	108.4 ± 7.3	94.6 ± 5.9	61.7 ± 10.0
	Chloroform	103.5 ± 17.3	40.5 ± 9.0	4.0 ± 0.8	1.3 ± 0.4
	Insoluble ethyl acetate	110.6 ± 13.7	125.1 ± 15.9	115.5 ± 13.3	121.3 ± 14.2
	Soluble ethyl acetate	108.3 ± 12.5	113.8 ± 13.8	121.9 ± 12.7	99.7 ± 15.6
	*n*-butanol	107.3 ± 11.9	120.8 ± 10.3	119.3 ± 12.3	113.6 ± 14.0
	Aqueous	45.9 ± 7.7	41.4 ± 7.8	48.8 ± 7.5	62.0 ± 7.9
**Leaves**	Methanol	84.8 ± 8.4	111.5 ± 4.3	115.6 ± 8.1	91.6 ± 6.3
	*n*-hexane	119.7 ± 3.6	129.0 ± 5.5	125.5 ± 7.9	121.4 ± 4.7
	Chloroform	89.0 ± 9.5	36.9 ± 8.3	1.1 ± 0.2	2.4 ± 0.5
	Ethyl acetate 1	115.5 ± 7.2	120.9 ± 8.0	113.5 ± 7.0	87.5 ± 6.1
	Ethyl acetate 2	65.5 ± 14.9	89.3 ± 15.9	105.2 ± 10.8	103.1 ± 6.9
	*n*-butanol	94.6 ± 11.8	93.4 ± 13.7	100.7 ± 13.4	110.0 ± 13.2
	Aqueous	56.1 ± 12.4	63.6 ± 11.4	61.4 ± 12.8	81.3 ± 10.6

**Table 4:** Effect of extracts prepared from *T. chrysantha* on the proliferation of *T. gondii* in HFF-1 cells.

Part of the Plant	Extract	Percentage of Viability (Mean ± SEM)			
		1 µg/mL	5 µg/mL	10 µg/mL	20 µg/mL
**Inner bark**	Methanol	95.8 ± 5.2	125.4 ± 6.6	139.3 ± 10.6	132.2 ± 9.2
	*n*-hexane	109.2 ± 3.3	115.1 ± 3.3	106.5 ± 5.9	70.1 ± 8.9
	Chloroform	129.1 ± 2.5	100.8 ± 2.2	48.8 ± 8.8	6.9 ± 0.7
	Soluble ethyl acetate	110.9 ± 10.8	136.2 ± 15.6	127.6 ± 13.5	99.9 ± 13.6
	Insoluble ethyl acetate	98.1 ± 11.6	126.4 ± 12.1	141.0 ± 14.3	138.9 ± 13.4
	*n*-butanol	65.6 ± 8.1	98.6 ± 13.4	100.0 ± 15.5	97.7 ± 14.7
	Aqueous	89.0 ± 14.0	97.0 ± 11.9	98.6 ± 12.7	82.6 ± 8.4
**Leaves**	Methanol	82.8 ± 6.8	106.8 ± 10.1	82.3 ± 5.2	41.2 ± 7.4
	Chloroform	93.3 ± 5.2	57.9 ± 9.8	23.5 ± 7.3	3.6 ± 0.4
	Ethyl acetate	93.2 ± 12.0	112.9 ± 8.1	116.6 ± 8.5	113.2 ± 9.2
	*n*-butanol	104.4 ± 14.0	91.0 ± 11.4	101.5 ± 8.7	85.4 ± 12.3
	Aqueous	60.4 ± 9.1	79.2 ± 7.3	88.4 ± 8.6	86.3 ± 8.8

**Table 5:** Effect of promising extracts on the proliferation of *Toxoplasma gondii* in HFF-1 cells.

Treatment	CC_50_^a^ (μ g/mL)	IC_50_^b^ (μ g/mL)	TI ^c^
Chloroform extract of *T. rosea* leaves	50	8.15	6.13
Chloroform extract of *T. rosea* inner bark	>50	2.91	>17.18
Chloroform extract of *T. chrysantha* leaves	>50	7.92	>6.31
β-amyrin	15.1	4.75	3.18

^a^ CC_50_—Cytotoxicity concentration of 50%. ^b^ IC_50_—Mean inhibitory concentration, a measure of the inhibition of tachyzoites. ^c^ TI—Therapeutic index, a measure of efficacy, calculated as CC_50_/IC_50_.

## Data Availability

The data presented in this study are available on request from the corresponding author.

## Supplementary Materials

The following supporting information can be downloaded at: https://www.mdpi.com/article/10.3390/molecules29050920/s1. Supplementary information Cardona et al.
