# Supplementary material for: In Vitro Anti-Toxoplasma Activity of Extracts Obtained from Tabebuia rosea and Tabebuia chrysantha: The Role of β-Amyrin
Source: Molecules. 2024 Feb 20;29(5):920. doi: 10.3390/molecules29050920 (PMC10933876; doi:10.3390/molecules29050920)

Supplementary information Phytochemical analysis

Table S1. Preliminary phytochemical analysis of extracts prepared from the inner bark of *T. rosea*

PHYTOCHEMICAL COMPONENT	REAGENT	MEOH	HEX	$CHCL_3$	ACET (SUN)	ACET (INS)	BUOH	H_2O
**PHENOLS AND TANNINS**	$FeCL_3$/EtOH	+	-	++	+	+	+	-
**FLAVONOIDS**	$AlCl_3$/EtOH	+++	+	+	+++	+++	+++	+
**LIGNANS**	UV 365 nm	++	-	+	++	++	++	+
	Vanillin/EtOH-H_3PO_4	+++	+++	+++	+++	+++	+++	++
**ANTHRONES**	KOH/EtOH	++	-	-	-	++	++	++
**ANTHRAQUINONES**		+	-	+++	-	-	-	-
**COUMARINS**		++	++	+++	+++	+++	+++	+
**TERPENES/TERPENOIDS STEROLS/STEROIDS**	Liebermann-Burchard	++	+++	+++	+++	+++	++	+
	Vanillin-H_3PO_4	+++	++	+++	+++	++	++	++
**IRIDOIDS**	Anisaldehyde- AcAc-H_2SO_4	+	++	++	+	+	-	-
	Vanillin-H_2SO_4	++	+++	+++	+++	++	-	-
**TRITERPENES**	Anisaldehyde- AcAc-H_2SO_4	-	-	-	-	-	-	-
**SAPONINS**		++	+	+++	++	++	++	-
**TRITERPENES AND SAPONINS**	$SbCl_3$/MeOH	++	+++	+++	+++	++	++	-
**SESQUITERPENE LACTONES**	Oleum	-	-	-	-	-	-	-
**ALDEHYDES AND KETONES**	DNPH	++	++	+++	+	++	++	+
**UNSATURATED FATTY ACIDS**	Iodine	+++	+++	+++	+++	+++	+++	++
**ANTIOXIDANT COMPOUNDS**	DPPH	+++	+++	+++	+++	+++	+++	+++

Table S2. Preliminary phytochemical analysis of extracts obtained from *T. rosea* leaves

PHYTOCHEMICAL COMPONENT	REAGENT	MEOH	HEX	$CHCL_3$	ACET (SUN)	ACET (INS)	BUOH	H_2O
PHENOLS AND TANNINS	$FeCL_3$/EtOH	++	-	-	+++	+++	+++	-
FLAVONOIDS	$AlCl_3$/EtOH	++	+	-	+++	+++	++	+
LIGNANS	UV 365 nm	+	-	-	++	++	++	+
LIGNANS	Vanillin/EtOH-H_3PO_4	++	++	++	+++	++	++	++
ANTHRONES	KOH/EtOH	+++	+	++	+++	+++	+++	+
ANTHRAQUINONES	KOH/EtOH	-	-	-	-	-	-	-
COUMARINS	KOH/EtOH	+	+	-	+++	++	++	+
TERPENES/TERPENOIDS STEROLS/STEROIDS	Liebermann-Burchard	+++	+++	+++	+++	++	+++	+
TERPENES/TERPENOIDS STEROLS/STEROIDS	Vanillin-H_3PO_4	+++	+++	+++	+++	+++	+++	-
IRIDOIDS	Anisaldehyde-AcAc-H_2SO_4	+++	+++	+++	++	-	-	-
IRIDOIDS	Vanillin-H_2SO_4	+++	+++	+++	+++	-	++	-
TRITERPENES	Anisaldehyde-AcAc-H_2SO_4	++	+++	+++	+	-	-	-
SAPONINS	Anisaldehyde-AcAc-H_2SO_4	+++	+++	+++	++	++	++	-
TRITERPENES AND SAPONINS	$SbCl_3$/MeOH	+++	+	++	+++	+++	+++	-
SESQUITERPENE LACTONES	Oleum	-	-	-	-	-	-	-
ALDEHYDES AND KETONES	DNPH	++	+++	++	+++	++	++	+
UNSATURATED FATTY ACIDS	Iodine	++	+++	+++	+++	++	+++	++
ANTIOXIDANT COMPOUNDS	DPPH	+++	+++	+	+++	+++	+++	++

Table S3. Preliminary phytochemical analysis of extracts prepared from the inner bark of *T. chrysantha.*

PHYTOCHEMICAL COMPONENT	REAGENT	MEOH	HEX	$CHCL_3$	ACET (SUN)	ACET (INS)	BUOH	H_2O
**PHENOLS AND TANNINS**	$FeCL_3$/EtOH	++	+	+	+++	+++	+++	-
**FLAVONOIDS**	$AlCl_3$/EtOH	++	+	++	+++	+++	+++	-
**LIGNANS**	UV 365 nm	++	-	+	+++	++	++	-
	Vanillin/EtOH-H_3PO_4	+++	+	+	++	++	++	++
**ANTHRONES**	KOH/EtOH	++	+	-	+++	+++	+++	-
**ANTHRAQUINONES**		+	++	+++	-	-	-	-
**COUMARINS**		++	+	++	++	++	++	+
**TERPENES/TERPENOIDS STEROLS/STEROIDS**	Liebermann-Burchard	+++	+++	+++	+++	+++	+++	++
	Vanillin-H_3PO_4	++	+++	+++	+++	+++	+++	++
**IRIDOIDS**	Anisaldehyde-AcAc-H_2SO_4	++	+++	+++	+++	-	-	-
	Vanillin-H_2SO_4	+++	+++	+++	+++	+++	+++	+
**TRITERPENES**	Anisaldehyde-AcAc-H_2SO_4	++	+++	+++	++	-	-	-
**SAPONINS**		++	+++	++	++	-	-	-
**TRITERPENES AND SAPONINS**	$SbCl_3$/MeOH	+	+++	+++	++	++	++	+
**SESQUITERPENE LACTONES**	Oleum	-	-	-	-	-	-	-
**ALDEHYDES AND KETONES**	DNPH	++	+++	+++	++	++	++	-
**UNSATURATED FATTY ACIDS**	Iodine	+++	+++	+++	+++	+++	+++	++
**ANTIOXIDANT COMPOUNDS**	DPPH	+++	+++	+++	+++	+++	+++	+++

Table S4. Preliminary phytochemical analysis of extracts prepared from leaves of *T. chrysantha.*

Phytochemical Core	Reagent	MeOH	CHCl3	AcEt	Buoh	H_2O
Phenols and Tannins	$FeCL_3$/EtOH	++	-	+++	+++	-
Flavonoids	$AlCl_3$/EtOH	+++	-	+++	+++	++
Lignans	UV 365 nm	+++	+	+++	+++	++
	Vanillin/EtOH-H_3PO_4	+++	+	+++	+++	++
Anthrones	KOH/EtOH	++	+	+++	+++	++
Anthraquinones		-	-	+	-	-
Coumarins		+	+	+	++	+
Terpenes/Terpenoids Sterols/Steroids	Liebermann-Burchard	+++	+++	++	++	++
	Vanillin-H_3PO_4	++	+++	+++	+++	++
Iridoids	Anisaldehyde- AcAc-H_2SO_4	+++	+++	++	-	-
	Vanillin-H_2SO_4	+++	+++	+	+	-
Triterpenes	Anisaldehyde- AcAc-H_2SO_4	+++	+++	+	-	-
Saponins		+++	+++	++	++	+
Triterpenes Saponins	$SbCl_3$/MeOH	++	++	++	++	+
Sesquiterpene Lactones	Oleum	-	-	-	-	-
Aldehydes and Ketones	DNPH	+	-	+++	++	++
Unsaturated Fatty Acids	Iodine	+++	+++	+++	+++	+++
Antioxidant compounds	DPPH	+++	++	+++	+++	+++

Supplementary information β-amyrin spectra

RMN-¹H β-amirin

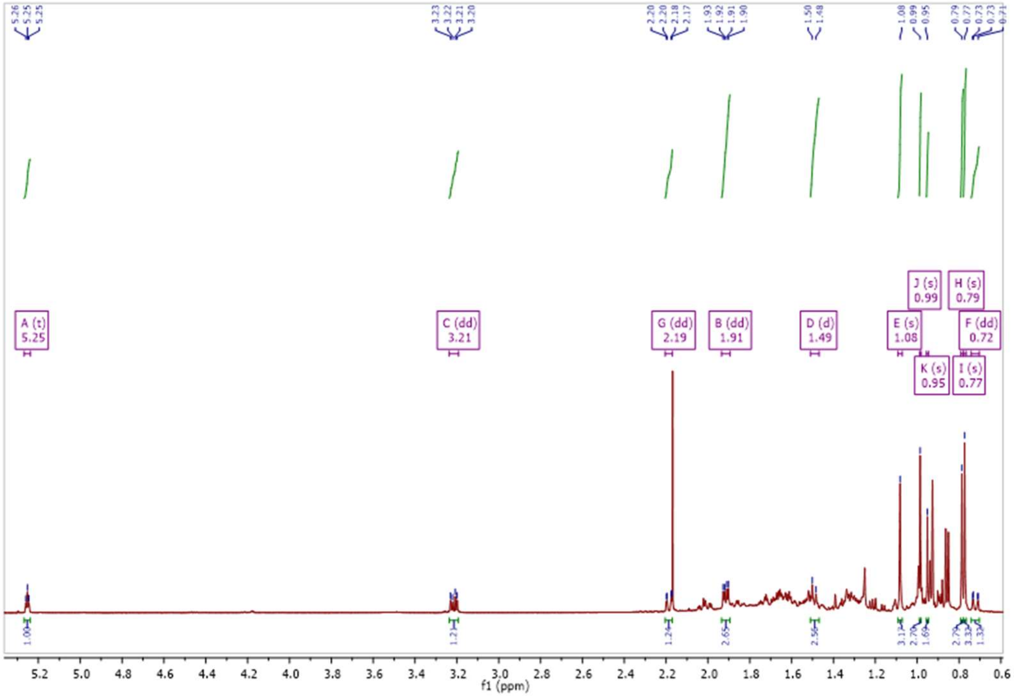

RMN-^{13}C β-amirin

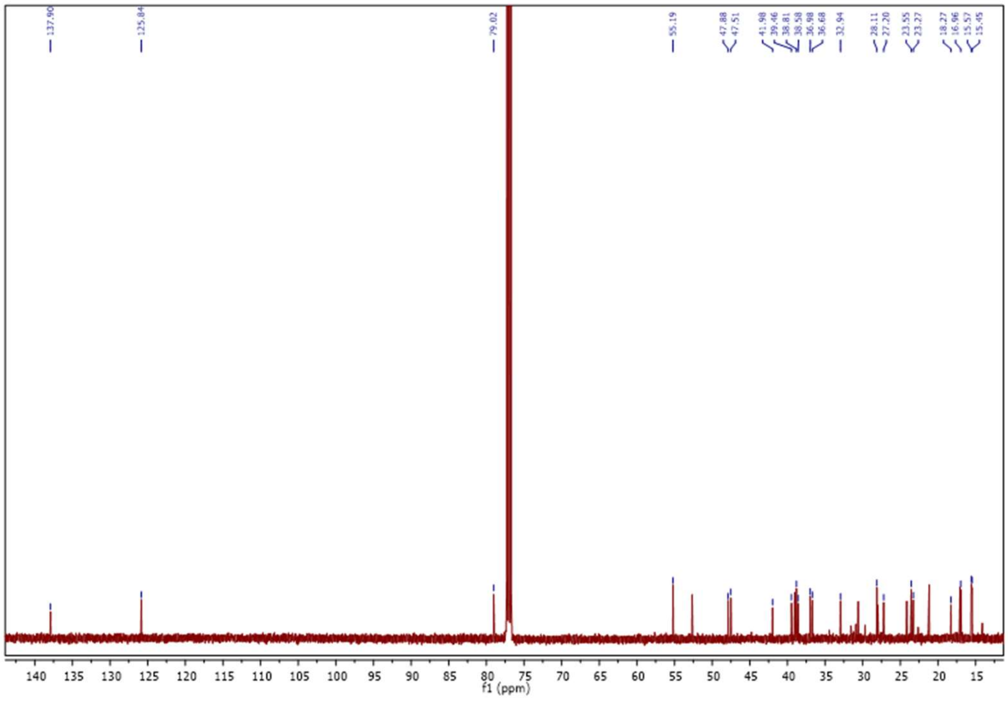

COSY β-amirin

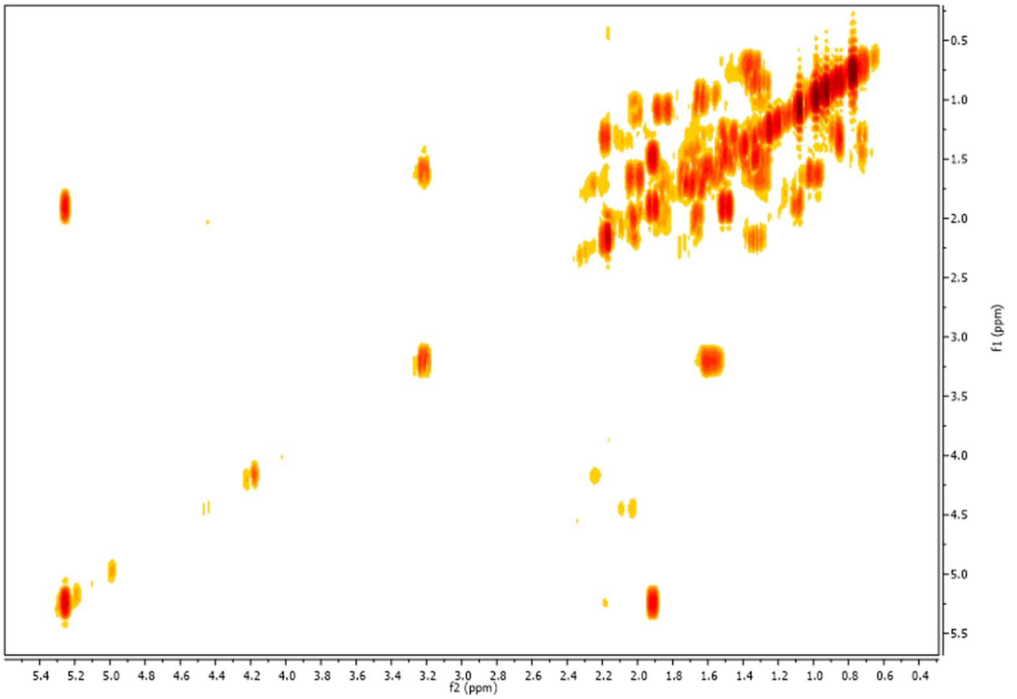

HSQC β-amirin

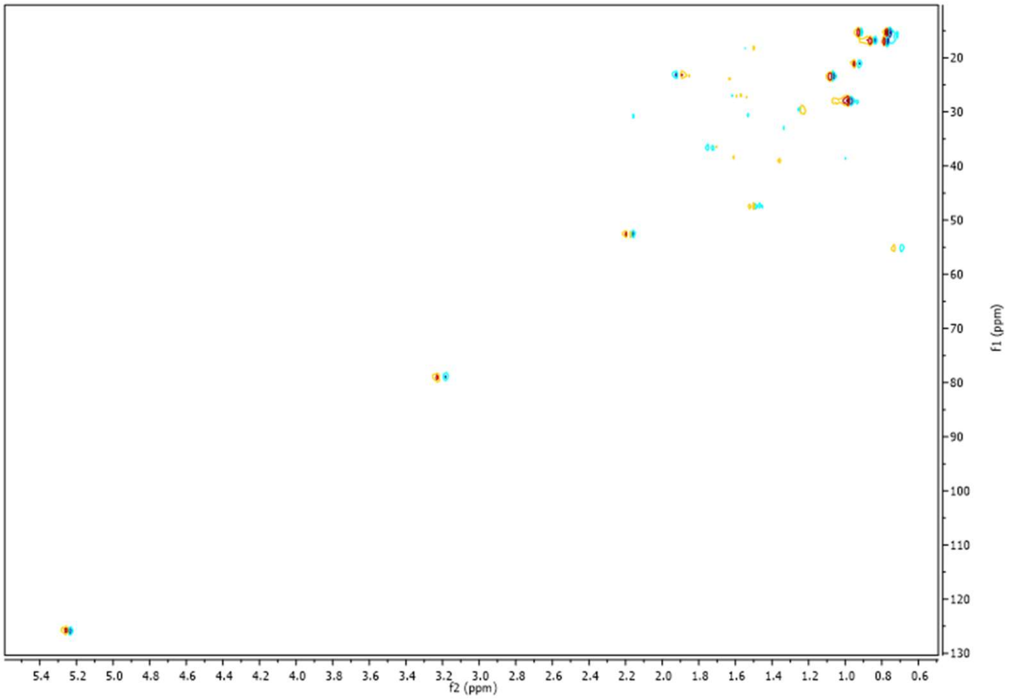

HMBC β-amirin

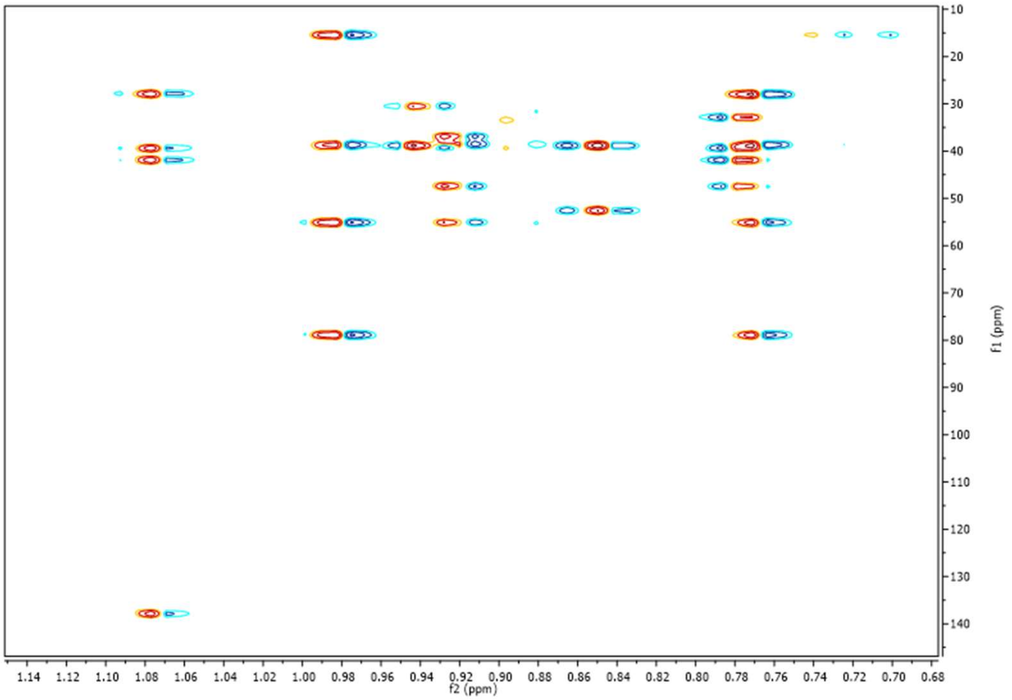

Supplement: Supplementary file 1 [file molecules-29-00920-s001.zip › molecules-2825549-supplementary.pdf]
